# Hydroxytyrosol and Arginine as Antioxidant, Anti-Inflammatory and Immunostimulant Dietary Supplements for COVID-19 and Long COVID

**DOI:** 10.3390/foods12101937

**Published:** 2023-05-10

**Authors:** José Manuel Pérez de la Lastra, Celia María Curieses Andrés, Celia Andrés Juan, Francisco J. Plou, Eduardo Pérez-Lebeña

**Affiliations:** 1 Institute of Natural Products and Agrobiology, CSIC-Spanish Research Council, Avda. Astrofísico Fco. Sánchez, 3, 38206 San Cristóbal de la Laguna, Spain; 2 Hospital Clínico Universitario of Valladolid, Avenida de Ramón y Cajal, 3, 47003 Valladolid, Spain; cmcurieses@gmail.com; 3 Cinquima Institute and Department of Organic Chemistry, Faculty of Sciences, Valladolid University, Paseo de Belén, 7, 47011 Valladolid, Spain; celia.andres.juan@uva.es; 4 Institute of Catalysis and Petrochemistry, CSIC-Spanish Research Council, 28049 Madrid, Spain; fplou@icp.csic.es; 5 Sistemas de Biotecnología y Recursos Naturales, 47625 Valladolid, Spain; info@glize.eu

**Keywords:** arginine, hydroxytyrosol, innate and adaptive immunity, COVID-19 and long COVID

## Abstract

Phytochemicals from plant extracts are becoming increasingly popular in the world of food science and technology because they have positive effects on human health. In particular, several bioactive foods and dietary supplements are being investigated as potential treatments for chronic COVID. Hydroxytyrosol (HXT) is a natural antioxidant, found in olive oil, with antioxidant anti-inflammatory properties that has been consumed by humans for centuries without reported adverse effects. Its use was approved by the European Food Safety Authority as a protective agent for the cardiovascular system. Similarly, arginine is a natural amino acid with anti-inflammatory properties that can modulate the activity of immune cells, reducing the production of pro-inflammatory cytokines such as IL-6 and TNF-α. The properties of both substances may be particularly beneficial in the context of COVID-19 and long COVID, which are characterised by inflammation and oxidative stress. While l-arginine promotes the formation of ^•^NO, HXT prevents oxidative stress and inflammation in infected cells. This combination could prevent the formation of harmful peroxynitrite, a potent pro-inflammatory substance implicated in pneumonia and COVID-19-associated organ dysfunction, as well as reduce inflammation, improve immune function, protect against free radical damage and prevent blood vessel injury. Further research is needed to fully understand the potential benefits of HXT and arginine in the context of COVID-19.

## 1. Introduction

Humanity is always exposed to the emergence of new pathogens that can compromise its economy and survival, as was the case with the pandemic caused by the SARS-CoV-2 virus [1]. According to the WHO (https://covid19.who.int/), accessed on 1 April 2023, 762 million people worldwide have had COVID-19 and the number of deaths is 6,893,190, with the highest number of reported cases and deaths in the United States of America [2]. The current coronavirus disease (COVID-19) is classified as a primary respiratory and vascular disease that can cause endothelial dysfunction (ED), acute lung injury (ALI), and acute respiratory distress syndrome (ARDS) in extreme situations [3]. The mortality rate among COVID-19 survivors is relatively low, but some people develop persistent symptoms that doctors called “long COVID” or “post-COVID syndrome” (PCS) [4]. Long-term COVID is characterized by a constellation of respiratory, cardiovascular, gastrointestinal, and neurological signs and symptoms, including dyspnea, fatigue, cardiac arrhythmias, heartburn, and memory and concentration problems (sometimes known as “brain fog”) with a substantial impact on quality of life [5]. Several mechanisms involved in the pathogenesis of long COVID are currently being explored, including viral persistence, chronic inflammation, autoimmune disease, metabolic and redox balance disturbances, and endothelial dysfunction [6]. A study on patients with COVID-19, who were followed up to 6 months after the disease, found that approximately 30% reported persistent post-COVID symptoms [7]. Although the development of long-term symptoms following SARS-CoV-2 infection may seem new or surprising, it is in fact an expected phenomenon. A majority of the viral or bacterial pathogens already studied were associated with the development of chronic symptoms in a subset of patients who have been infected [8]. These patients suffer from prolonged COVID or post-acute sequelae (PASC) of COVID-19 [9,10].

Some PASC patients meet the diagnostic criteria for those suffering from myalgic encephalomyelitis/chronic fatigue syndrome (ME/CFS), a form of neuroinflammation characterized by chronic debilitating symptoms such as severe fatigue, musculoskeletal pain, and discomfort following physical exertion or exercise [10,11]. The delay in developing specific treatments for long COVID is due to heterogeneity inthe clinical symptoms, so treatment has focused primarily on symptomatic medications and healthy lifestyle suggestions [12].

We here review the role of two natural compounds that could help to reduce the severe symptoms of COVID-19 and reduce or shorten long COVID: a well-known polyphenol, hydroxytyrosol (HXT), characterised by its antioxidant and anti-inflammatory activity, and the amino acid arginine. There are two main reasons why we decided to focus on HXT. First, HXT is easily absorbed by the human body due to its basic composition. Secondly, the European Food Safety Authority has approved HXT as a means of protecting the cardiovascular system. From a theoretical point of view, we study the interaction between HXT and arginine to stop the production of harmful peroxynitrite, which is responsible for pneumonia and numerous organ failures associated with COVID-19 [13].

## 2. Plant Polyphenols and Flavonoids—Importance of the Concept of Bioavailability in the Function of Polyphenols

There are three types of phenols, which can be distinguished by their chemical composition: polyphenols (which include tannins and flavonoids), simple phenols (which include phenolic acids), and a third class that includes everything else [14]. Polyphenols are a category of plant-based chemical compounds characterized by the presence of multiple phenolic units or building blocks per molecule [15]. Antioxidants such as polyphenols can help prevent or neutralize free radical damage when it occurs [16,17]. They also impart colour to edible plants such as flowers, fruits, and vegetables. Polyphenols are capable of modulating biological events in different diseases, based on their antioxidant and anti-inflammatory capacity. Electrostatic interactions, hydrogen bonds, and ionic bonds between phenolic hydroxyl groups and the positively charged amino groups in proteins are possible. Targeting enzymatic sources of ROS without affecting the physiological redox state is an important purpose [18,19].

In general, any phytochemical with a 15-carbon (C6-C3-C6) backbone is called a flavonoid, and more than 4500 different flavonoids have been isolated from plants. They can be classified into several subgroups, such as flavones, flavanones, flavonols, dihydroflavonols, isoflavonoids aurones, chalcones, biflavonoids, etc. [20]. Olive plants contain three primary phenolic chemicals: oleuropein (OLE), hydroxytyrosol (HXT), and tyrosol [21,22]. Oleuropein, a heterosidic ester of β-glucosylatedelenolic acid and 3,4-dihydroxy-phenylethanol, is derived from the secondary metabolism of terpenes and belongs to the secoiridoids, and it is found in concentrations of up to 140 mg/g in young olives and 60 mg/g in their leaves (in dry weight basis, in both cases) [23]. In both olive fruit and the human body, HXT and tyrosol are formed as byproductsfrom the breakdown of oleuropein. After consumption, the oleic acid in the fruit is hydrolyzed during maturation and processing and then further processed to hydroxytyrosol by lipase activity. The HXT content in extra virgin olive oil (EVOO) and table olives can be influenced by a number of variables, including the region where the olives were grown, the plant species used, the harvest date, and the processing methods used [23]. The HXT concentration in EVOO is 14.32 ± 3.01 mg/kg, while in refined virgin olive oil, it is only 1.74 ± 0.84 mg/kg. Spanish green olives have an HXT content of 170–510 mg/kg, Greek black olives of 100–340 mg/kg, and Greek Kalamata olives of 250–760 mg/kg [24]. HXT found in olive oil is an extremely potent natural antioxidant, twice as effective as coenzyme Q10. Some classes of chemicals containing amino groups are structurally attractive to HXT. The basic structure of HXT ensures that it is readily absorbed by the human body. Within 15 to 20 min of absorption, it reaches the plasma without causing toxicity problems [25,26]. Since it can easily cross cell membranes, this carrier is very effective for transporting substances through the body. The advantageous structural and transport properties of HXT at the molecular level contribute to a wide range of beneficial effects on the organism [25]. In 2012, HXT was approved by the European Food Safety Authority as a protective natural agent for the cardiovascular system [27].

Polyphenols are gaining increasing interest, particularly because of the link between their consumption and the prevention of various diseases including cardiovascular disorders, cancer, neurodegenerative dysfunctions, diabetes, and others [28]. Although there is a link between polyphenol consumption and reduced risk of chronic diseases, it should be appreciated that the discrepancies and contradictions obtained in different studies in explaining this possible beneficial effect are based on their bioavailability [29].

Several clinical analyses on the use of polyphenols have limitations, such as small numbers of participants, lack of controls, use of different methods, and heterogeneity in the types of correlations in the databases [30]. Other studies have been conducted on a broader basis and were well-designed (such as PREDIMED). This analysis showed that the Mediterranean diet has a lower cardiovascular risk and improves cognitive function in the elderly. The beneficial effects of this study may be due to the water solubility of hydroxytyrosol and oleuropein [31].

In addition to the importance of bioavailability on health effects, the average daily intake should also be taken into account. A recent systematic review of the literature, which included more than 90 human studies, estimated the intake of polyphenols based on dietary habits and identified an average daily intake of polyphenols in the general population (including adolescents, adults, and the elderly) of 0.9 g/day, the main sources being coffee, tea, red wine, fruits, and vegetables. This intake was associated with a reduction in cardiovascular disease (CVD) and type 2 diabetes mellitus [32].

Polyphenols are more soluble in organic solvents (less polar) than in water, and the solubility is dependent on the polar properties of the polyphenols. HXT has a water solubility of 50 g/L. In addition, HXT has a bioavailability of 99%, so it is easily integrated by the human body [33] and is resistant to gastric juices as it passes through the digestive system and is recovered in the urine mainly in the form of 3’-sulphate [34].

## 3. Antiviral Properties of Hydroxytyrosol

The antiviral properties of secondary metabolites found in plants are well documented [35]. These compounds are known to affect all stages of the virus life cycle (host contact, entry, replication, assembly, and release). Sometimes, polyphenols interfere with the attachment of a virus to host cells by binding to the viral capsid (envelope). Hydroxytyrosol is particularly effective against enveloped viruses such as influenza, human immunodeficiency virus, and coronavirus [36,37].

The antiviral activities of HXT were studied by Kentaro Yamada and colleagues with influenza A virus, New Castle disease virus, bovine rotavirus, and chicken adenovirus [36]. Although HXT proved successful in combating enveloped viruses, it proved ineffective with non-enveloped viruses. For example, the structure of the H9N2 virus was altered and surface spines were absent when the virus was treated with HXT, as observed using electron microscopy. These results indicate that the viral envelope is the primary target of HXT. The H9N2 virus treated with HXT showed antiviral properties, including a reduction in mRNA production and the absence of viral nucleoproteins [36].

In a study using an animal model, the HXT of olive oil showed anti-inflammatory effects [38]. HXT decreased the expressions of pro-inflammatory cytokines such as TNF and IL-1 in inflammatory disorders. HXT suppressed the expression of inducible nitric oxide synthase (iNOS), cyclooxygenase (COX-2), and tumour necrosis factor (TNF-α) in LPS-challenged human monocytic THP-1 cells in vitro. HXT has been shown to minimize the oxidative damages caused by inflammation by inhibiting the AA-derived lipoxygenase (LOX) and cyclooxygenase enzymes [39]. Because of its anti-inflammatory effects, HXT has been considered a non-toxic drug for inflammation regulation.

The nuclear factor NF-κβ signalling pathway is activated by numerous discrete stimuli and is a master regulator of the inflammatory response to pathogens and cancerous cells, as well as a key regulator of autoimmune diseases.NF-κβ signalling is crucial for the development and activation of adaptive immune cells [40,41,42]. NF-κβ occupies a vital upstream position in the inflammatory cascade, where it controls the production of countless proinflammatory mediators. ROS (primarily H_2_O_2_ and ^•^O_2_^−^) [43] can modulate intracellular signalling pathways, activating the NF-κβ factor. Enrico Sangiovanni et al. evaluated the effect of olive oil phenols on NF-κβ activity in human gastric adenocarcinoma cells [44]. Olive oil phenol extracts inhibited NF-κβ in a concentration-dependent manner: IC(50) for the Italian and the Spanish extract were 0.86 and 1.28 µg/mL, respectively. The IC(50) for individual compounds ranged from 4.5 to 13 µM. These extract compounds inhibited nuclear translocation as well. The data suggest that consumption of extra-virgin olive oil may be beneficial for preventing the onset of inflammation leading to more serious diseases [44].

Hydroxytyrosol is also a powerful anti-inflammatory compound. The secretion of cytokines (IL-1, IL-1, IL-6, IL-12, and TNF-α) and chemokines (CXCL10/IP-10 and CCL2/MPC-1) was reduced by effectively inhibiting the production of ^•^NO and prostaglandin E2 (PGE2) in a study highlighting this property [45]. Reducing iNOS and COX-2 gene expression, as well as blocking NF-kβ, the signal transducer and activator of transcription 1 (STAT-1), and the interferon regulatory factor (IRF-1) activation are all effects of HXT therapy [46].

**Figure 1:**
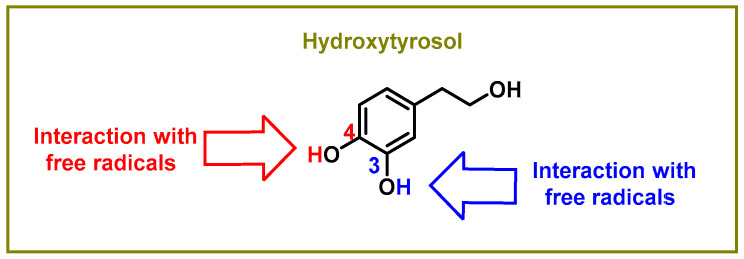
Sites of the interaction between the hydroxytyrosol molecule and free radicals.

The hydroxyl groups on the aromatic rings in HXT have the capacity to donate an H+ to a variety of radicals, including superoxide anion, hydroxyl, peroxyl, etc., which lose reactivity as a result of stabilization, creating a r a relatively stable radical [47], as shown in Figure 1. Superoxide anion is the first by-product of O_2_ reduction [48]. Classically, it has a dual role, as it can induce cell death pathways, apoptosis, necrosis, ferroptosis, pyroptosis, and autophagic cell death. However, at the same time, it is involved in physiological processes and is beneficial for the oxidative death of microbial pathogens, which are subsequently engulfed by specialised immune cells, such as neutrophils or macrophages, during the activation of the innate immune system [49].

**Figure 2:**
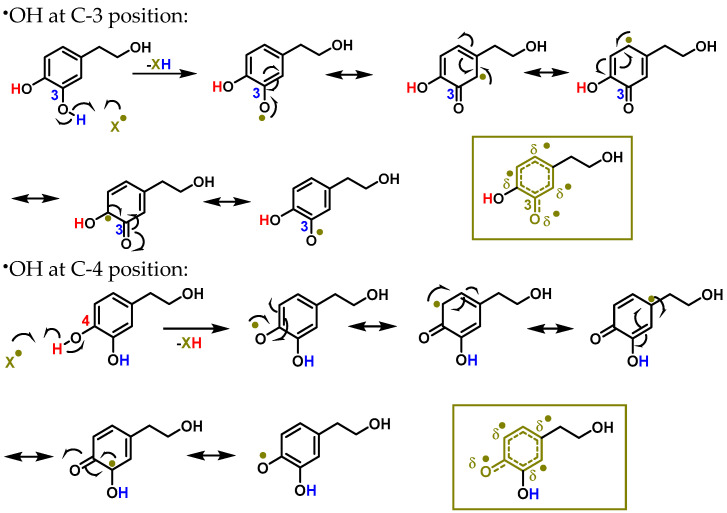
The interaction between hydroxytyrosol (HXT) and a free radical ^•^X at the hydrogen in the OH of C-3 and C-4 positions results in electron delocalisation throughout the aromatic ring.

The delocalisation of electrons throughout the aromatic ring is caused by an interaction with a free radical ^•^X at the hydrogen in the OH of the C-3 and C-4 positions, as shown in Figure 2. An oxygen, nitrogen, or chlorine radical, such as hydroxyl, peroxyl, superoxide, or peroxynitrous acid, can be the ^•^X radical [47].

Scientists have discovered that this chemical regulates the redox state in cells and prevents oxidative stress from damaging cells. Vilaplana-Pérez et al., 2014, examined the properties of HXT and its mechanisms of action, which include potent antioxidant and anti-inflammatory effects, among others [27]. They highlighted the importance of HXT in the protection of low-density lipoproteins and, consequently, its involvement in reducing the risk of cardiovascular disease, which was also highlighted by the European Food Safety Authority. They concluded that 5 mg of HXT and its derivatives should be consumed daily to achieve this effect at the physiological level [27].

Physiological and pathological inflammatory responses are associated with the production of cytokines (TNF-α, IL -1, IL -6, and IL -17), chemokines, and adhesion molecules.Another study found that hydroxytyrosol affected the activation of the transcription factor NF-kB. SIRT1 activation increased the effect of HXT on endothelial nitric oxide synthase (eNOS) phosphorylation in glucose-stimulated human umbilical vein endothelial cells (HUVECs). However, the promoting effect of HXT on eNOS phosphorylation was abolished by the deactivation of sirtuin SIRT1. Most importantly, HXT inhibited the production of reactive oxygen species (ROS) via SIRT1 in HUVECs. The ROS scavenger potentiated the effect of HXT on eNOS phosphorylation [50].

Shan and Miao, 2022, evaluated the immunomodulatory and antioxidant effects of HXT in immunosuppressed broilers. Immunosuppressed broiler models were established with intraperitoneal injection of 80 mg/kg cyclophosphamide. The HXT groups were treated with 0.5 mL of 200 mg/L HXT solution, once daily, for 7 days. HXT was found to increase the villus height (HV)/crypt depth (CD) ratio in the duodenum and suppressed serum levels of TNF-α and interleukin-6 (IL-6). In addition, it elevated CD4+ and CD8+ T-lymphocyte expression. HXT increased the mRNA expression levels of IL-2, IL-4, and IL-10, increased the activity of superoxide dismutase (SOD) and glutathione peroxidase (GSH-Px), and reduced the levels of malondialdehyde (MDA) in the cyclophosphamideCy-induced immunosuppressed broilers. They concluded that HXT alleviates immunosuppression, as well as improves immunity and antioxidant activities in the local small intestinal mucosa of broiler chickens. Therefore, it was proposed that HXT can be used as an immune stimulant [51].

These properties have earned hydroxytyrosol much attention as a potential agent to defend against COVID-19 infections [52,53].

Arginase catalyses the last step in the urea cycle, a series of biochemical reactions in which the mammalian organism eliminates harmful ammonia.However, arginase activity is increased during inflammatory processes and with reactive oxygen species associated with disease states. Although plant polyphenolic compounds belonging to the dihydroxyphenyl group show little inhibitory effect on arginase and are not specific, they still exhibit antioxidant activity and potentially cardiovascular protective effects [54].

## 4. The Importance of Amino Acids in the Immune Response

In their simplest form, amino acids were the building blocks for life on Earth. The combination of amino acids to form peptides and eventually proteins that mediate biological functions was a crucial evolutionary step [55]. Before cellular biosynthesis evolved, only a select group of 10 amino acids may have been used for protein synthesis [56]. In nature, there are many more amino acids that affect cellular function, but mammals use only a subset of 20 for protein synthesis [57,58]. Nine of the twenty amino acids used to make proteins are considered “essential” because the body cannot make enough of them on its own. In contrast, the body can make sufficient amounts of the non-essential amino acids. Thus, they are not needed but may become temporarily necessary if their demand exceeds their supply [59,60].

Amino acids are important not only for protein production but also for a variety of actions that promote growth and cell division. Protection against infection and tolerance to harmless environmental antigens are normal results of the immune response [60]. The vertebrate immune system is divided into innate and adaptive immune systems, which were once considered independent but are now known to be closely linked through the release of cytokines and chemokines. The establishment of an efficient immune response depends on the interaction between and regulation of innate and adaptive immunity [61].

The metabolic status of immune cells is critical because their activation status is constantly changing in response to infections and changes in their tissue environment. Immune cells rely on amino acids for adequate nutrition, and amino acid availability controls immune cell activity. Immune cells have specific amino acid requirements. During an immune response, non-essential amino acids can become conditionally essential as immune cells undergo dramatic changes in cell growth and proliferation in response to changes in their extracellular environment [60]. Amino acids stored in lysosomes can be released or restricted as needed to trigger autophagy as a protective mechanism during times of nutrient scarcity [62]. Stimulation by growth factors and activation of T cells drives their rapid proliferation, which in turn increases the need for an increased amino acid supply [63]. Abnormal proliferation and polarization of Th1, Th2, Th9, Th17, or Th22 cell populations are hallmarks of the chronic proinflammatory state that results from an active immune response in many diseases [64,65]. Withtheir involvement in central energy metabolism, redox balance, epigenetic modifications, and post-translational modifications (PTMs), amino acids can influence immunity through multiple mechanisms [60]. Different immune cells are thought to respond differently to amino acid perturbations when an immune cell expresses transporters and internal metabolic enzymes to process the amino acids [65]. Viruses have adapted to specifically target amino acid transporters because amino acid uptake is an early checkpoint for manipulating amino acid metabolism. Targeting amino acid metabolism in immune cells is a useful technique to enhance or antagonize immune responses [66,67]. Many new amino acid metabolic pathways remain to be studied for their potential effects on immune cells, and a better understanding of amino acid metabolism in immune cells should be of great therapeutic benefit [66]. Therefore, the identification of appropriate regulatory factors that promote protective immune responses, while limiting tissue-damaging immune responses, is a critical prerequisite for the development of novel therapeutics [68,69].

## 5. Role of ^•^NO in the Immune Response against SARS-CoV-2

**Figure 3:**
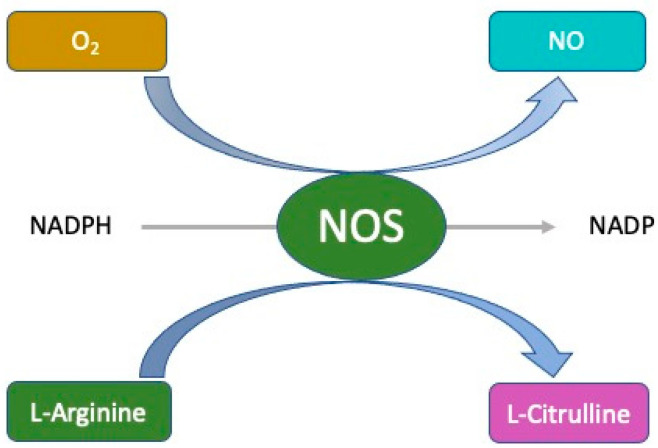
The biological mechanisms by which nitric oxide is produced in living organisms.

The SARS-CoV-2 virus damages multiple cell types (alveocytes, macrophages, T cells, etc.), resulting in a massive release of proinflammatory cytokines (a “cytokine storm”). When this happens, nitric oxide (^•^NO), one of the main mediators of local inflammation, is reduced by inhibiting the formation of all types of nitric oxide synthases (NOSs) [70,71]. Nitric oxide is a free radical gas that acts as a messenger throughout the body. This tiny chemical, previously thought to be an air pollutant, was transferred into biological organisms by pharmacology professor Lou Ignarro, where it has vasodilatory effects, among others [72]. The enzyme NOS catalyses the production of ^•^NO in cells (Figure 3) that typically express ACE2 receptors, including epithelial cells, endothelial cells, macrophages, neutrophils, mast cells, and T lymphocytes [73,74].

The vasodilator, bronchodilator, antiapoptotic, antiplatelet, antioxidant, and antiproliferative properties of ^•^NO in physiological doses (5–10 nmol) are well documented [75]. Under the influence of ^•^NO, cyclic guanylate cyclase is strongly expressed in airway smooth muscle cells, resulting in a bronchodilator effect. Inducible nitric oxide synthase (iNOS) is found in macrophages and tissues, whereas endothelial nitric oxide synthase (eNOS) is found in the endothelium of organs. NOS2/iNOS was first described as an enzyme produced in activated macrophages that produces nitric oxide from the amino acid l-arginine, which helps regulate the replication or death of intracellular microbial pathogens [74,75]. High expression of NOS2 is thought to be mainly restricted to the adaptive phase of the immune response, as interferon (IFN)-gamma is the essential cytokine for stimulating NOS2 in macrophages and the prototypical product of type 1 T helper cells [76]. Apoptosis in myeloid inflammatory cells is mainly controlled by ^•^NO [77]. Macrophage cells release ^•^NO in response to inflammatory signals, and an increase in ^•^NO in macrophages leads to various alterations in macrophage immunometabolism with far-reaching effects in COVID-19 patients [78]. Mononuclear cells, which include macrophages, produce proinflammatory cytokines; supplementation with the ^•^NO precursor arginine in COVID-19 patients reduces this production [79].

The protective effect of ^•^NO is consistent with the extent of inhibition in viral replication. As demonstrated by Akaberi et al., inhaled ^•^NO gas appeared in the midst of the COVID-19 pandemic as a promising therapeutic modality with the potential to improve patient oxygenation, leading to improved clinical outcomes [80]. However, using inhalation of ^•^NO as a treatment has many limitations because it may lead to methemoglobinemia, which in turn reduces the oxygen-carrying capacity of the blood [81,82]. Another important problem with current ^•^NO gas therapies is that ^•^NO can be degraded in the presence of oxygen into nitrogen dioxide (NO_2_), a toxic gas that has ^•^NO therapeutic benefit [75]. Long-term exposure to NO_2_ can cause oedema, bronchoconstriction, and a decrease in forced expiratory volume in the lungs [83]. In hospitalized cases of COVID-19, the efficacy of existing ^•^NO gas therapy protocols has been questioned. Depending on the context, endogenous nitric oxide can have either helpful or harmful effects on the cardiovascular and other systems [84]. Excessive nitric oxide production by iNOS is associated with cardiovascular dysfunction, bioenergetic failure, and cellular toxicity in sepsis [85] (a syndrome that has many clinical and pathophysiological similarities to COVID-19), whereas decreased nitric oxide synthesis by the endothelium may contribute to impaired microvascular function [70].

## 6. Arginine Metabolism in the Immune Response against SARS-CoV-2

The SARS-CoV-2 virus enters the body by binding to angiotensin-converting enzyme 2 (ACE2), which is highly expressed in the human endothelial cells lining the arterial and venous systems [86,87]. Endothelial cells play a critical role in preventing the spread of the virus. Endothelial cell dysfunction is associated with the pathological consequences of COVID-19 [88]. Recent metabolomics research shows that l-arginine metabolic pathways are altered in COVID-19 patients and that peripheral blood mononuclear cells (PBMCs) from COVID-19 patients have increased levels of arginase mRNA [89]. Arginase causes endothelial dysfunction by degrading l-arginine and reducing the bioavailability of ^•^NO, and it is likely to be associated with COVID-19 and its vascular consequences [90]. SARS-CoV-2 infection of the endothelium leads to the loss of the vasodilator ^•^NO [90]. It is known that ^•^NO, an unconventional messenger molecule, controls vascular smooth muscle proliferation, regulates vasodilation, and inhibits platelet aggregation and adhesion. The suppression of ^•^NO in the endothelium caused by SARS-CoV-2 ED leads to thrombosis and endotheliitis, followed by vasoconstriction and tissue hypoxia, thus restoring ^•^NO improves lung function [91].

l-arginine is a semi-essential amino acid that has antiviral and immunomodulatory properties and promotes the formation of ^•^NO in endothelial cells. The upregulation of arginase activity that occurs during acute COVID-19 causes immunologic and endothelial dysfunction, inflammation, and thrombosis by lowering circulating l-arginine levels and diverting metabolism from generating ^•^NO [92]. Indeed, lower plasma levels of l-arginine and increased arginase activity were found in both patients with COVID-19 and children with multisystem inflammatory syndrome (MIS-C) [93]. Acute COVID-19 has also been associated with a decrease in plasma l-arginine levels, which has been linked to myeloid suppressor cell growth and decreased T-cell proliferation, two characteristic inflammatory aspects of severe disease [94]. Arginine promotes CD4+ and CD8+ T cell survival by triggering a switch in metabolism to oxygen consumption and increasing free respiratory capacity in activated T cells [95]. To ensure a steady supply of metabolites, macrophages take up arginine from apoptotic cells, where it is processed by arginase I and activates the actin-regulatory protein Rac1 in a putrescine-dependent manner [96] (macrophages take up arginine and ornithine from apoptotic cells during efferocytosis, the process by which apoptotic cells are removed by phagocytic cells, and putrescine augments subsequent rounds of efferocytosis by increasing Rac1 activation).

In cancer, inflammation, and bacterial and viral infections, a heterogeneous population of cells called myeloid-derived suppressor cells (MDSCs) proliferate beyond normal physiological regulation. While the main role of polymorphonuclear MDSCs is to dampen the response of T cells and NK killer cells, they also produce ROS and reactive nitrogen species (RNS) [97].

In addition to inhibiting NK cell activity and IFN-γ production, MDSCs reduce arginine supply to T cells by increasing arginine metabolism via arginase [98]. Decreased NK cell survival, expression of NK cell-activating receptors NKp46 and NKp30, and IFN-γ production are all effects of arginine deficiency [99]. In chronic hepatitis C infection, the virus takes advantage of this mechanism to dampen the body’s natural antiviral defences [100]. Similarly, arginase ARG1 activity is increased during Th1 polarization. Large numbers of G-MDSC cells, which can consume l-arginine and cause poor T-cell receptor and endothelial function, have also been detected in the lungs of patients who died from problems related to COVID-19 [79]. To address the immunologic, pulmonary, and vascular consequences of COVID-19, techniques to restore circulating l-arginine levels and improve ^•^NO bioavailability have been proposed [79,101]. Patients with severe COVID-19 who took oral l-arginine in addition to their normal medication spent significantly less time in the hospital, according to a clinical trial [102]. Concomitant intake of l-arginine and vitamin C also reduced the severity of long-term symptoms COVID-19 [103,104].

## 7. Role of l-arginine and ^•^NO in SARS-CoV-2 Infection

**Figure 4:**
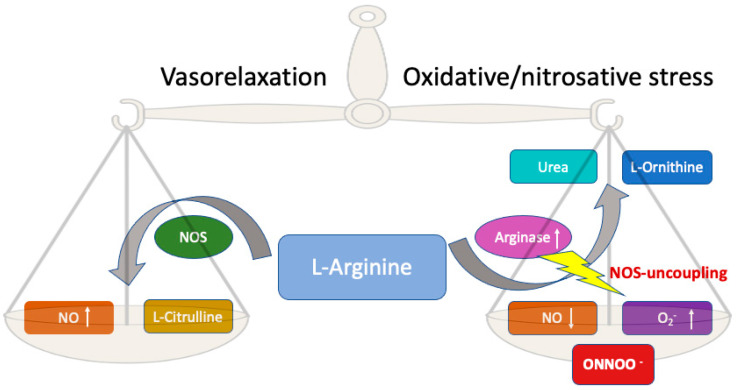
The central role of l-arginine in the biological production of nitric oxide (^•^NO). l-arginine is converted by arginase into urea and l-ornithine. At the same time, l-arginine is a substrate for the enzyme NOS, which converts l-arginine into L-citrulline and ^•^NO, which can improve vasorelaxation. When arginase levels increase, NOS uncoupling may occur, resulting in lower ^•^NO production and higher superoxide anion (^•^O_2_^−^) and peroxynitryte (ONOO^−^) levels.

Arginase is a ubiquitous manganese metalloenzyme with l-arginine hydrolase activity that can convert l-arginine into l-ornithine and urea and regulates the production of vasodilatory nitric oxide (^•^NO) in the endothelium by competing with eNOS for the substrate l-arginine [105,106]. Specifically, an increase in arginase activity may lead to the uncoupling of eNOS, thereby decreasing the production of ^•^NO and increasing the generation of reactive oxygen species (ROS) [70,107]. The conversion of l-arginine to L-citrulline yields nitric oxide and reactive nitrogen intermediates such as peroxynitrite [108]. The overproduction of reactive oxygen species (ROS) and a deficiency of nitric oxide both contribute to the development of endothelial dysfunction. Increasing oxidative stress exacerbates the uncoupling of NOS. This leads to “nitrosative stress”, which, together with “oxidative stress”, aggravates the progression of the “cytokine storm” [108]. Nitric oxide is required for the synthesis of many reactive oxygen and nitrogen species, including peroxynitrite, nitrous oxide, and nitrogen dioxide, all of which can inhibit viral replication [109]. l-arginine may reduce peroxynitrite formation and suppress the release of proinflammatory cytokines from alveolar macrophages [110]. Various viruses and bacteria are known to be either blocked or stimulated by higher levels of ^•^NO, and it is also known that iNOS is often upregulated during infection. Both l-arginine release and ^•^NO antagonize SARS-CoV-2 infection in different ways (via antiviral and immunomodulatory mechanisms, respectively) [70,107]. Considering the central role that ^•^NO and l-arginine play in maintaining proper endothelial function, an imbalance between arginase and NOS, caused by the overexpression of arginase, may lead to excessive l-arginine consumption, resulting in a lack of substrate for NOS to form ^•^NO [70,107]. The uncoupling of NOS induced by decreased bioavailability of l-arginine leads to the production of superoxide anions instead of ^•^NO and enhances the inactivation of ^•^NO [108](Figure 4). 

Therefore, l-arginine is important for the protection against cardiovascular disease and the maintenance of endothelial function [108].

## 8. Hydroxytyrosol and Arginine for COVID-19

Understanding the mechanisms for viral entry into the body, the pathophysiologic and pathomorphologic manifestations, and the likely post-disease consequences are important parts of solving the difficult and multidimensional problems posed by the global COVID-19 pandemic. For a better understanding of disease treatment and prevention, it is essential to initially propose hypotheses based on theoretical arguments and then to access empirical tests and trials.

The proposal presented in this manuscript focuses on the administration of two natural compounds that can help relieve the severe symptoms of COVID-19 and shorten or reduce long COVID. They consist of the co-administration of the amino acid arginine, to ensure continuous production of ^•^NO, and a well-known polyphenol, hydroxytyrosol (HXT), for three reasons [111]: (i) it is water soluble (5 g/100 mL), which is necessary for its absorption and complete diffusion in the body [47]; (ii) it is a polyphenol characterised by its antioxidant and anti-inflammatory activity (the latter is of real interest in the context of the cytokine storm); and (iii) the ability of polyphenols to inhibit viral replication is well known. HXT exhibits antiviral properties againstthe influenza A (IAV) and human immunodeficiency (HIV) viruses [36,37]. Similar to IAV and HIV, SARS-CoV-2 is a single-stranded RNA virus with an envelope; therefore, it is to be expected that HXT is alsocapable of inhibiting SARS-CoV-2 replication.

Related to COVID-19 and acute respiratory distress syndrome (ARDS), but probably in other viral infections, there is a low arginine availability, and this may also lead to eNOS uncoupling, which induces further oxidative and cellular damage in the pulmonary epithelium and endothelium [112,113,114]. High levels of ^•^NO are detrimental because, in the presence of the viral infection and the subsequent oxidising state in which high amounts of ^•^O_2_^−^ are generated, peroxynitrite is produced [109]. In order to reduce the peroxynitrite load, it is important to administer an antioxidant such as HXT and/or polyphenols in general.

HXT is a polyphenol that is at the centre of the so-called Mediterranean paradox, characterised by the levels of cardiovascular morbidity and mortality from cardiovascular risk factors [115,116]. HXT is the only polyphenol on the market that supports a health claim by the European Food Safety Authority [38,117]. The Mediterranean diet is based on the consumption of fruits, vegetables, and olive oil. Although people in Mediterranean countries tend to consume relatively high levels of fat, they have much lower rates of cardiovascular disease than in countries such as the United States, where fat consumption is similarly high.

The European Commission has asked the Scientific Panel on Dietetic Products, Nutrition and Allergies of the European Food Safety Authority (EFSA) for an opinion on HXT [118] as a novel food. In its response, HXT was found to comply with the requirements for novel food ingredients in Article 3(1) of Regulation (EC) 258/97 and to be safe for use by the general public, with the exception of children under three years of age and pregnant or lactating women. With a limit for unobserved adverse effects of 50 mg/kg body weight per day, HXT is considered safe by the EFSA as a novel food for human consumption [118]. In the United States, 5 mg of HXT per serving is considered a safe amount for use in processed foods [119,120]. Therefore, an adult dose of 800 mg per day is safe.

For most adults, a dose of up to 30 g of l-arginine per day is safe, according to a 2018 study on 142 participants. The study participants tolerated 15–30 g of the supplement without experiencing negative side effects [121]. They conclude that a safe amount of L-Arg supplementation for adults in the long term is at least 30 g per day. Therefore, individuals can safely consume 5 g per day.

It is important to emphasize that more research is needed to properly understand the effects of hydroxytyrosol and arginine on COVID-19, although the theoretical analysis we performed for this publication provides some evidence for their potential benefits. Therefore, it would be premature to conclude that these supplements represent a cure for COVID-19 or even a surefire approach for improving health during or after infection until actual clinical trials are conducted.

## 9. Conclusions

The current treatment of COVID-19 requires the development of new and improved antiviral agents with unique mechanisms of action. Several bioactive foods and supplements are being investigated as potential treatments for chronic COVID. This article examines the potential roles of two specific compounds, arginine and hydroxytyrosol, in the context of COVID-19 and its long-term effects. l-arginine is an important modulator of endothelial, pulmonary, and immunological function. Nitric oxide synthase and arginase, two primary metabolizing enzymes, control its pleiotropic properties. Favourable effects on immunity and vascular health have been associated with its detour via l-arginine into ^•^NO, while its conversion into ornithine by the enzyme arginase is associated with aberrant immune responses and endothelial impairment. There is increasing evidence that people with COVID-19 have abnormal l-arginine metabolism. The upregulation of arginase activity during acute COVID-19 lowers circulating l-arginine levels and diverts metabolism from the formation of ^•^NO, eventually leading to arterial occlusion and multiple organ failure due to immunological and endothelial dysfunction, inflammation, and thrombosis. Hydroxytyrosol and l-arginine are two examples of supplements with antioxidant and anti-inflammatory effects that not only help the body fight viral infection but also improve immunity and prevent chronic fatigue and gastrointestinal problems in the period after COVID-19. Natural substances that have been shown to be safe, effective, and inexpensive are sought by scientists as a means of overcoming these obstacles. The chemical diversity and wide availability of natural products offer limitless promise for new drugs.

## Data Availability

Not applicable.
